# Supplementary figures and images for: A Simple, Cost-Effective, and Extraction-Free Molecular Diagnostic Test for Sickle Cell Disease Using a Noninvasive Buccal Swab Specimen for a Limited-Resource Setting
Source: Diagnostics (Basel). 2022 Jul 21;12(7):1765. doi: 10.3390/diagnostics12071765 (PMC9318149; doi:10.3390/diagnostics12071765)

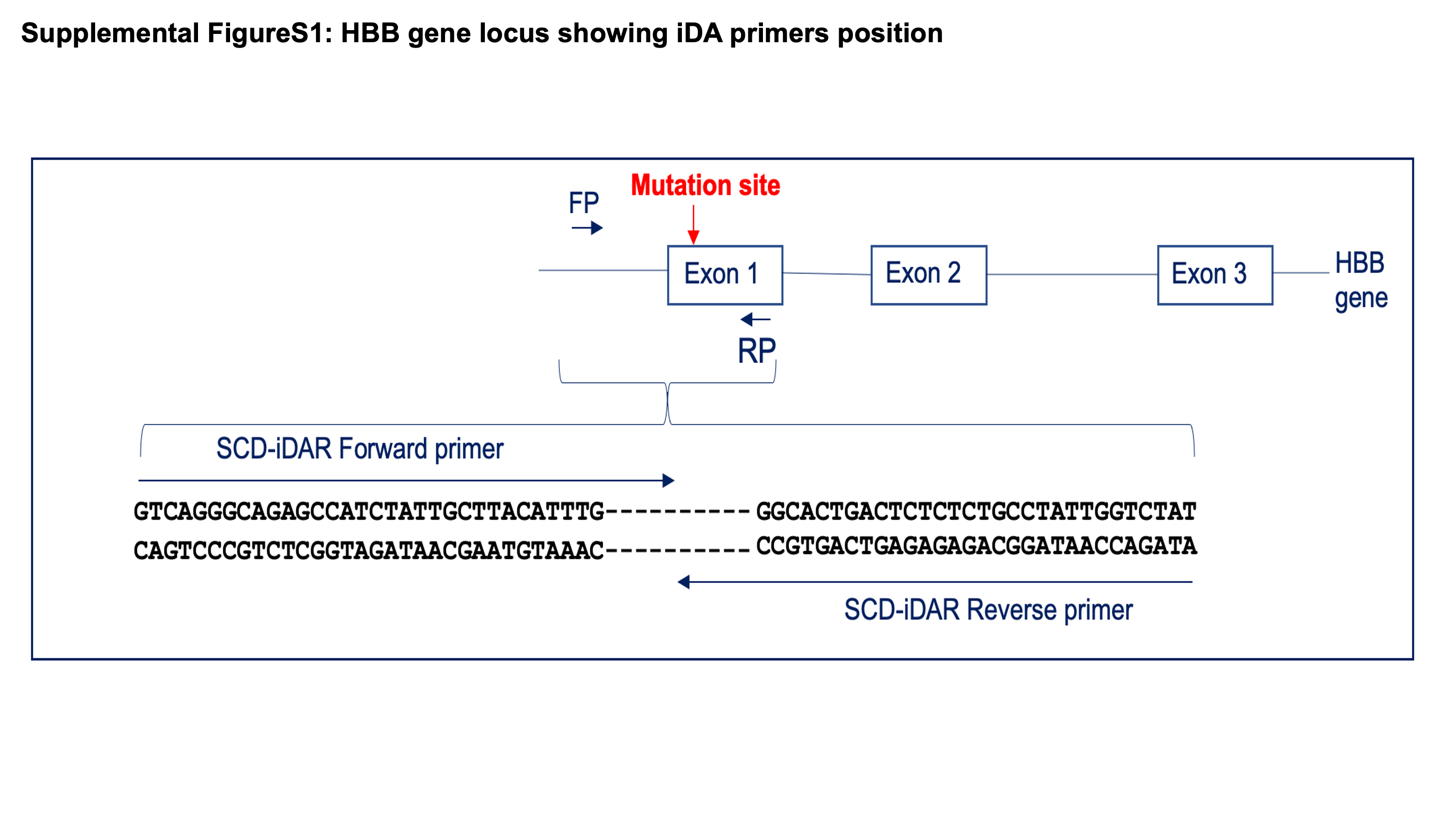

Supplement: Supplementary file 1 [file diagnostics-12-01765-s001.zip › Figure S1.tiff]

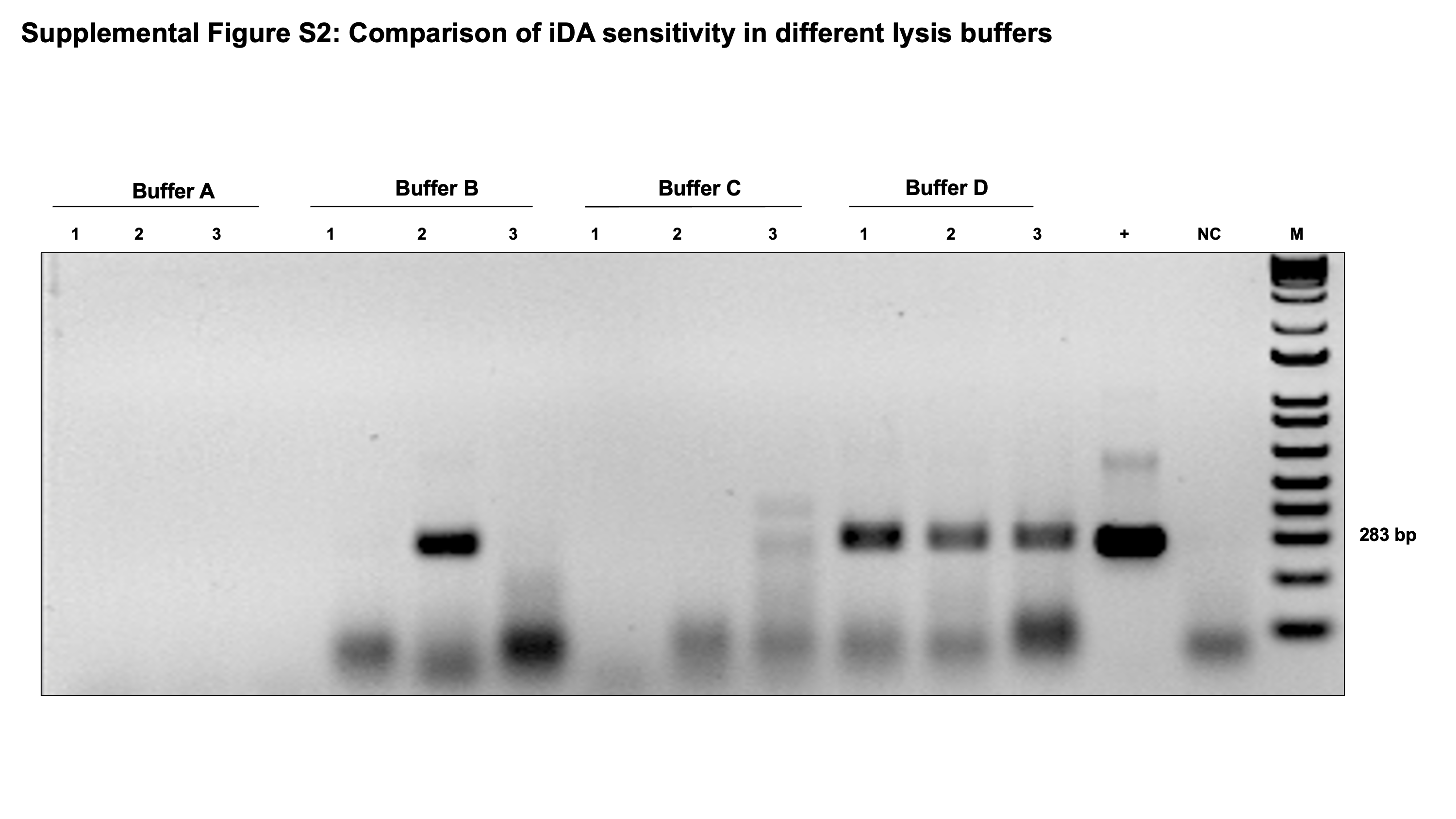

Supplement: Supplementary file 1 [file diagnostics-12-01765-s001.zip › Figure S2.tiff]

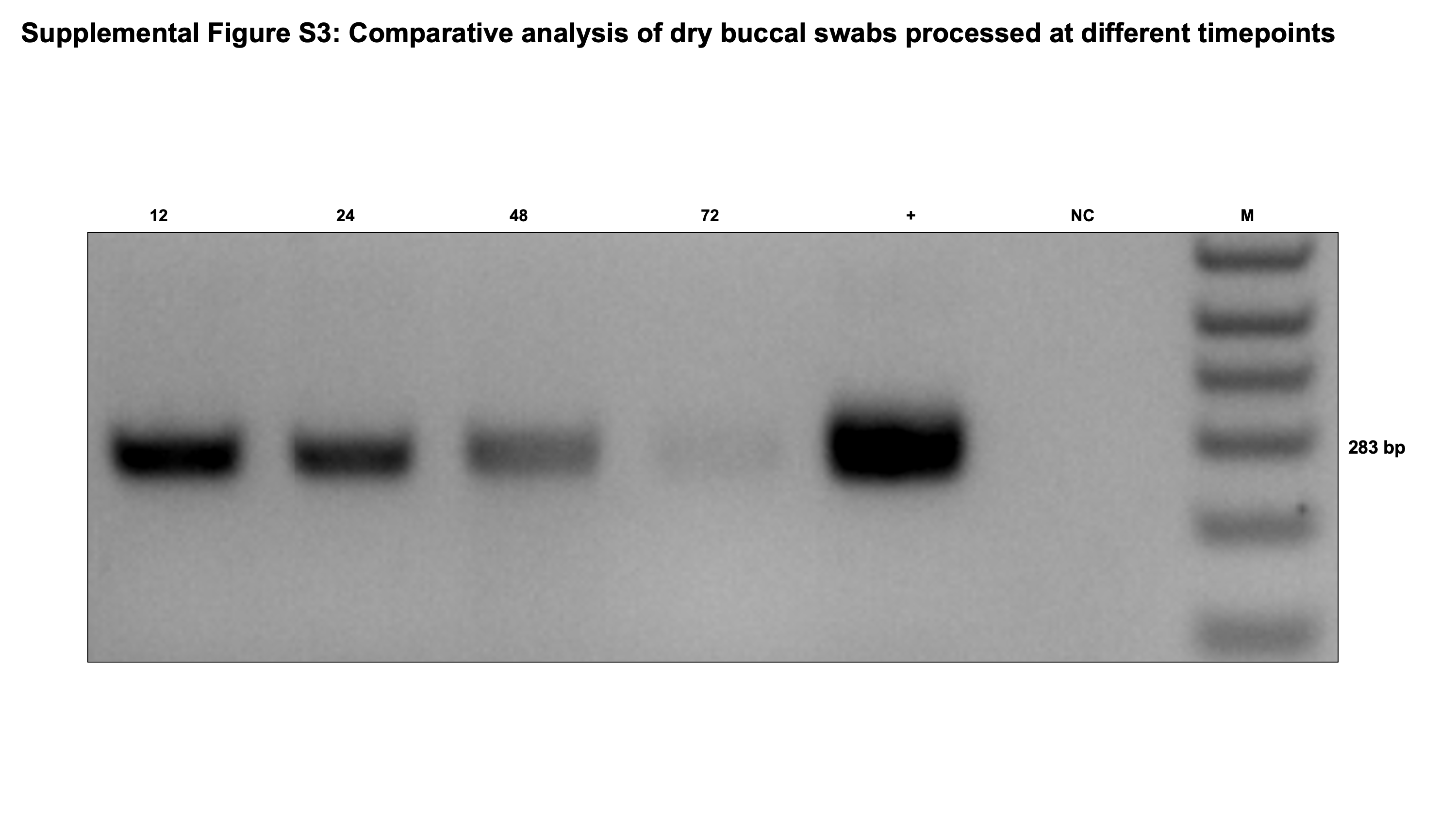

Supplement: Supplementary file 1 [file diagnostics-12-01765-s001.zip › Figure S3.tiff]

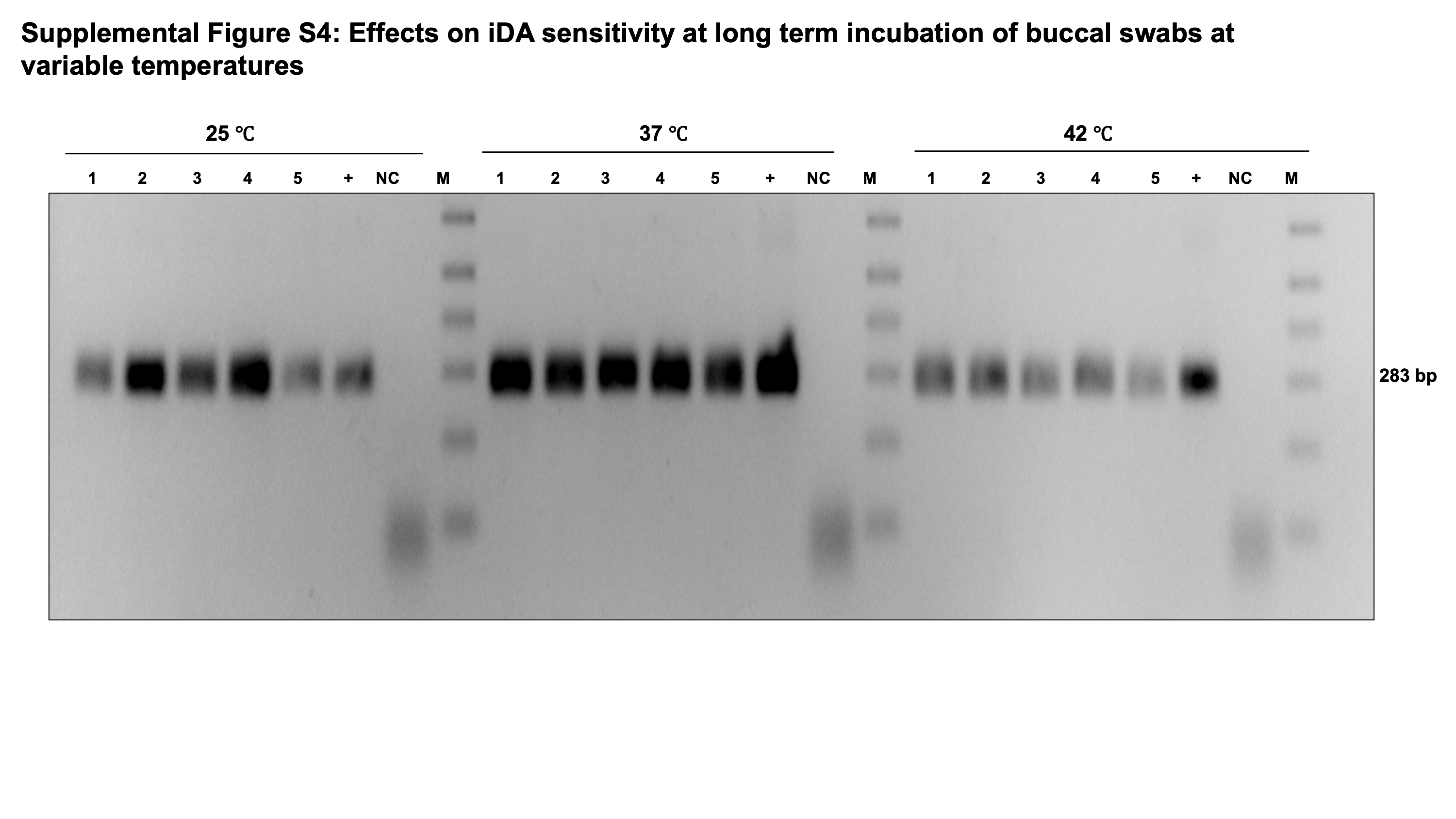

Supplement: Supplementary file 1 [file diagnostics-12-01765-s001.zip › Figure S4.tiff]
